# Supplementary material for: Increased mask adherence after important politician infected with COVID-19
Source: PLoS One. 2022 Jan 12;17(1):e0261398. doi: 10.1371/journal.pone.0261398 (PMC8754325; doi:10.1371/journal.pone.0261398)
Supplement: Attachment — Submitted filename: response to reviewers.docx [file pone.0261398.s001.docx]

**Response to reviewers:**

Thanks for your comments. First, RAND Corporation is NOT a commercial company. It is a non-profit research institute.

See other responses below.

Reviewer #1: The article presents a relevant and innovative theme. Some small adjustments must be made, they are:
- Improve the introduction, putting an international panorama on the theme (how is the relation of the use of masks in other countries)?

**We added Brazil as a country where mask adherence has been politicized.**

- Improve at the end of the discussion, what are the limitations of the study.

**We added additional limitations.**

- Create a paragraph at the end of the discussion with the practical / clinical implications of your study.

**We added this.**
- Improve completion by detailing in a topic. **Not sure what this means, but hope we provided sufficient detail.**

Reviewer #2: I am pleased to share my comment, for the article entitled: Increased Mask Adherence after President Trump Infected with COVID-19
Use the word leader or politicians instead of the names of people in the title.

**We changed to world leader.**

The necessity and importance of the study is not properly explained.

**The importance is due to prevention of spread of a deadly virus. Mask adherence is critical. Countries that have higher adherence have lower case rates.**

Why Philadelphia was studied.

**This was a matter of convenience, it is where our staff was located. We had instituted surveillance prior to President Trumps Covid-19 infection.**

What is the importance of the study results?

**The findings demonstrate the importance of messaging and media. People paid more attention when President Trump was infected than to health department warnings. Possibly giving examples and making the consequences more real maybe more effective.**

Who will benefit from the research results?

**Leaders, public health professionals and health care providers who want to increase adherence to public health guidance.**

In the introduction, use more studies and explain the importance of study.

**We added information on the importance of our study, but there are no similar studies that have used direct observation to monitor mask adherence.**

The method part should be described step by step and in more detail.

**We expanded.**

How was the correct use of the mask by people examined?

**By observation. Correct use was defined as covering both mouth and nose.**

Did people know they were being watched by the research team? **This is unknown. We had no interaction with those being observed.**

People are constantly moving and reorienting, how did you measure the appropriate social distance?

**This was a visual estimate.**

30 locations, why only parks, playgrounds and shopping streets? Are restaurants, passages (shopping malls) and entertainment centers less important?

**We stuck to outdoor locations for safety of the data collectors.**

How can you verify the accuracy of your observations?

**We conducted reliability testing. The results have been published and these are now referenced.**

How many days were the survey days? How many hours were observed each day?

**Each site was observed for one hour on the day and time of day over time.**

Were the weather conditions different between the review days and the days before?

**Yes, weather follows the seasons and the summer is typically warmer than the fall.**

Mention study limitations? **We added some more limitations.**

Conclusions should be based on the findings of the study. **We agree**

What are the benefits of the study for the health system?

**We added a paragraph on the implications.**

In the discussion section: in addition to describing the study and its important findings, Compare the findings with other studies and describe the solutions and challenges in this context.

**We are not aware of other published studies that have conducted serial observations of mask adherence. Nevertheless, there are multiple other studies employing direct observation that successfully document behavioral trends.**

Reviewer #3: Title: appropriate
Abstract: appropriate and adequate
Introduction: Authors have indicated the justification to do the study.
Methodology: It was not stated the number of adequate sample size for this research. The justification in choosing the location to be observed was not clearly expelled in the methodology. The characteristics of observers in the study were not clearly stated and various background may promotes bias that may affect the findings of the study. It must be addressed as limitation if there is.

**Because this is an innovative study there were no prior data informing sample size calculations.   Our sample size was based on three considerations.** **First, the number of observation locations was similar to our previous studies of direct observations of human physical activity behavior in built environment.  In many of our past studies, we usually selected anywhere between 6 to 50 sites (e.g., neighborhood parks, recreation centers), in a city for direct observations.   Second, the number of observed subjects needs to be sufficient to draw inference for the outcome of interest.   Since our outcome is a binary random variable in this paper, a total of 1000 or more subjects yielded sufficient power under the regular power setting of 2-sided p<.05 and power>.8 and for a small to medium effect size. As shown in Table 2, in retrospect we did have sufficient statistical power to declare significance for many substantive predictors.  Third, the sample size was also constrained by the available manpower we could deploy during the critical study period.  We were not able to further increase the number of locations given the available and trained observers.**

**We added this to the limitations.**

The SOMAD protocol showing that authors made attempt in standardising the research tool and data collection.
Results: appropriate
Discussion: the limitation of the study ie potential bias, limitation on generalisation of the findings were not discussed.

**We expanded the discussion of limitations.**
